# Evaluation of Physical Properties of Ti-Doped BiFeO_3_ Thin Films Deposited on Fluorine Tin Oxide and Indium Tin Oxide Substrates

**DOI:** 10.3390/ma18102395

**Published:** 2025-05-21

**Authors:** Anel Rocío Carrasco-Hernández, Armando Reyes-Rojas, Gabriel Rojas-George, Antonio Ramírez-De la Cruz, Hilda Esperanza Esparza-Ponce

**Affiliations:** 1 Facultad de Ciencias Químicas, Universidad Autónoma de Chihuahua, Circuito Universitario, Chihuahua 31125, Chihuahua, Mexico; arcarrasco@uach.mx (A.R.C.-H.); andelacruz@uach.mx (A.R.-D.l.C.); 2 Física de Materiales, Centro de Investigación en Materiales Avanzados S.C., Miguel de Cervantes 120, Chihuahua 31136, Chihuahua, Mexico; armando.reyes@cimav.edu.mx (A.R.-R.); gabriel.rojas@cimav.edu.mx (G.R.-G.)

**Keywords:** ferroelectrics, Ti-doped BiFeO_3_, sputtering

## Abstract

BiFeO_3_ is a fascinating material with a rhombohedral crystal structure (*R3c*) at room temperature. This unique structure makes it suitable for use in solar cells, as the interaction of light with the polarized octahedral enhances electron movement. Evaluating its properties on different substrates helps to identify the specific characteristics of thin films. The thin films presented in this work were deposited using reactive RF cathodic sputtering with a homemade 1-inch diameter ceramic target. Their morphology, phase composition, optical, piezoelectric, and ferroelectric properties were evaluated. Fluorine Tin Oxide (FTO) and Indium Tin Oxide (ITO) substrates were used for the presented thin films. The thin films deposited on FTO displayed the “butterfly” behavior typically associated with ferroelectric materials. A d_33_ value of 2.71 nm/V was determined using SSPFM-DART mode. In contrast, the thin films deposited on ITO at 550 °C reached a maximum saturation polarization of 40.89 μC/cm^2^ and a remnant polarization of 44.87 μC/cm^2^, which are the highest values recorded, but did not present the typical “butterfly” behavior. As the grain size increased, the influence of charge defects became more pronounced, leading to an increase in the leakage current. Furthermore, the presence of secondary phases also contributed to this behavior.

## 1. Introduction

Bismuth ferrite (BiFeO_3_) is a lead-free perovskite material with a rhombohedral crystal structure (*R3c*) at room temperature, lattice parameters *a* = 5.58 Å and *c* = 13.90 Å, and a rhombohedral angle of 90° [1,2,3]. It is known for its low cost, non-toxicity, and environmental friendliness [4,5]. BiFeO_3_ is the only multiferroic material that operates at room temperature, with a Curie temperature of 850 °C and a Néel temperature of 367 °C [4,6,7]. Its band gap (2.2–2.7 eV) [1,8] allows absorption of ~20% of the solar spectrum, making it a promising candidate for photovoltaic applications [1,9,10,11]. Other potential uses include photoelectrocatalysis, memory devices, spintronics, capacitors, and sensors [12,13].

Despite its potential, BiFeO_3_ synthesis faces challenges due to phase instability above 675 °C, leading to secondary phases [5,6,14] and defects such as bismuth vacancies [15] and iron valence fluctuations, which cause dielectric losses and leakage currents [4,12]. Doping with alkali or transition metals, such as titanium (Ti^4+^), has been shown to enhance the electrical properties and, consequently, the photovoltaic characteristics of the material, improving its power conversion efficiency [16]. Ti^4+^ is chosen due to its ionic radius (0.605 Å), which is similar to that of Fe^3+^ (0.645 Å), facilitating its substitution [17]. Additionally, Ti^4+^ incorporation reduces the concentration of oxygen vacancies and Fe^2+^ ions, which decreases dielectric loss. Substituting titanium ions at the B-site can also enhance magnetization by disrupting the balance between antiparallel spin networks of Fe^3+^ ions [4]. However, excessive dopant concentrations may lead to the formation of oxygen vacancies, negatively impacting the dielectric properties [12].

Several studies have explored the effects of doping on the physical properties of BiFeO_3_ thin films. These films have been deposited on various substrates using different deposition techniques. For instance, in 2015, Chen et al. deposited BiFeO_3_ thin films on ITO, FTO, and platinum (Pt) substrates using cathodic sputtering. They found that 195 nm-thick films deposited on ITO exhibited the highest remanent polarization (2Pr = 180 μC/cm^2^), up to seventy-three times greater than those deposited on FTO (2Pr = 2.5 μC/cm^2^) and Pt (2Pr = 63 μC/cm^2^), respectively. They concluded that ITO is the most suitable substrate for BiFeO_3_ thin film deposition [18]. In 2016, Rong et al. demonstrated improvements in the properties of titanium-doped BiFeO_3_ thin films with 0, 5, and 10% deposited on FTO substrates. Doping with 5% titanium led to a band gap reduction to 1.97 eV and a notable enhancement in absorption within the visible light spectrum. Additionally, doping enhanced the carrier concentration, leading to a considerable improvement in the photoelectrocatalytic performance. These findings underscore the crucial role of titanium in optimizing the electronic and photoelectrochemical properties of BiFeO_3_ [17]. In 2018, Kumari et al. investigated the photovoltaic performance of BiFeO_3_ thin films, both undoped and doped with titanium, nickel, and cadmium. Among these materials, the titanium-doped films exhibited significant improvements in their photovoltaic parameters, with an open-circuit voltage (V_oc_) of 0.77 V, a short-circuit current density (J_sc_) of 7519 μA/cm^2^, and a conversion efficiency (η) of 3.043%. These results were notably higher compared to the undoped films and those doped with nickel and cadmium, highlighting titanium doping as a key strategy for enhancing the photovoltaic performance of bismuth ferrite [9].

BiFeO_3_ thin films can be synthesized through various deposition techniques, including spin-coating, dip-coating, pyrolysis spray, cathodic sputtering, pulsed laser deposition (PLD), and chemical vapor deposition (CVD) [9,19,20]. Cathodic sputtering is highly compatible with modern electronics, offering high-quality epitaxial thin films [20]. Film properties depend on target phase stability, growth conditions, gas composition, chamber pressure, sputtering power, and substrate temperature [21].

In this study, BiFeO_3_ thin films were deposited onto glass, Indium Tin Oxide (ITO), and Fluorine Tin Oxide (FTO) substrates using RF cathodic sputtering. The deposition times were 180 and 300 min, employing a ceramic target (BiFe_0.90_Ti_0_._10_O_3_) synthesized in our laboratory. Following deposition, the films underwent ex-situ heat treatment at temperatures of 550 °C and 575 °C for 60 min. We investigated the effects of deposition time and heat treatment temperature on the structural, ferroelectric, and optical properties of the thin films.

## 2. Materials and Methods

Ti-doped BiFeO_3_ (BFTO) thin films were deposited via RF cathodic sputtering using a 1-inch diameter homemade ceramic target fabricated through the sol-gel technique. The target was manufactured by preparing powders using the sol-gel method using bismuth nitrate pentahydrate (Bi(NO_3_)_3_·5H_2_O, 99.99% purity, Sigma-Aldrich, Burlington, MA, USA), iron nitrate nonahydrate (Fe(NO_3_)_3_·9H_2_O, 99.99% purity, Sigma-Aldrich), ethylene glycol (C_2_H_6_O_2_, 99.99% purity, J.T. Baker, Morristown, NJ, USA), and titanium isopropoxide (C_12_H_28_O_4_Ti, 97.0% purity, Sigma-Aldrich) in a stoichiometric ratio of BiFe_0.90_Ti_0.10_O_3_. A 10% bismuth excess was added to mitigate bismuth volatilization throughout thermal treatments.

The powders were calcined at 600 °C for 2 h and pressed at 4 MPa for 16 s using a Desk Top Electromotion Presser (MTI). The resulting pellet was sintered at 870 °C for 90 min in a KSL1100X furnace.

Thin films were deposited onto three different types of substrates, namely, Corning Glass, Indium Tin Oxide (ITO: resistance = 20 Ω), and Fluorine Tin Oxide (FTO: resistance = 23 Ω), using Sputtering V3 (Cuernavaca, Morelos, México) System with an RF source operating at 50 W. Deposition times of 180 and 300 min were employed using argon and oxygen flow rates of 15 and 1.6 cm^3^/min, respectively. The substrates were continuously rotated at 3 rpm without substrate heating. After deposition, the films were subjected to ex-situ thermal annealing in atmospheric conditions at 550 °C and 575 °C for 60 min to promote crystallization. Finally, circular silver electrodes were deposited to evaluate the ferroelectric properties. The electrodes were fabricated using the screen-printing technique with a stainless-steel mask containing 200 μm in diameter circles.

All samples were labeled according to the substrate, heat treatment temperature, and deposition time, following the experimental conditions shown in Table 1. Samples deposited on glass were not labeled. They are referenced separately, indicating the substrate and deposition time.

The crystalline structure of the thin films deposited on ITO and FTO was analyzed by X-ray diffraction (XRD) using a PANalytical X’pertPRO (Almelo, The Netherlands) diffractometer with Cu Kα radiation (λ = 0.15406 nm) in grazing incidence mode. Measurements were performed at room temperature over a 2θ range of 20–60°, with a step size of 0.05° and 9 s/step. The surface morphology of the thin films deposited on ITO and FTO and the thickness of those deposited on glass were analyzed using a JEOL JSM-7410F (Akishima, Tokyo, Japan) scanning electron microscope (FESEM). Atomic force microscopy (AFM) in tapping mode was performed using an Oxford Instruments (Abingdon, Oxfordshire, England) MFP-3D Infinity microscope to evaluate the topography, surface roughness, and grain size of thin films deposited on ITO and FTO over scanned areas of 10 × 10 μm and 1 × 1 μm. Domain switching was characterized by switching spectroscopy piezo response force microscopy—Dual AC Resonance Tracking mode (SSPFM-DART mode) to obtain amplitude and phase plots. All measurements were conducted in the “Off” state to clarify if any electrostatic interactions affect the true ferroelectric character of the thin films [22]. The measurements are normally presented in this state since this eliminates the long-range electrostatic force influencing the electromechanical response on the samples [23]. Absorbance spectra were obtained at room temperature using a Thermo Scientific (Waltham, MA, USA) UV-visible spectrometer ranging from 200 to 800 nm. Thin films deposited on glass were used to avoid ITO and FTO substrate contribution to the spectra. The optical band gap was estimated from Tauc plots based on the Kubelka–Munk theory, which describes the behavior of light within scattering samples. Ferroelectric hysteresis loops were measured using a Radiant Technologies P-PPM2 (Albuquerque, NM, USA) system under room temperature conditions at 10 kHz, with varying applied voltage, on thin films deposited on ITO and FTO.

## 3. Results and Discussion

### 3.1. X-Ray Analysis

**Figure 1:**
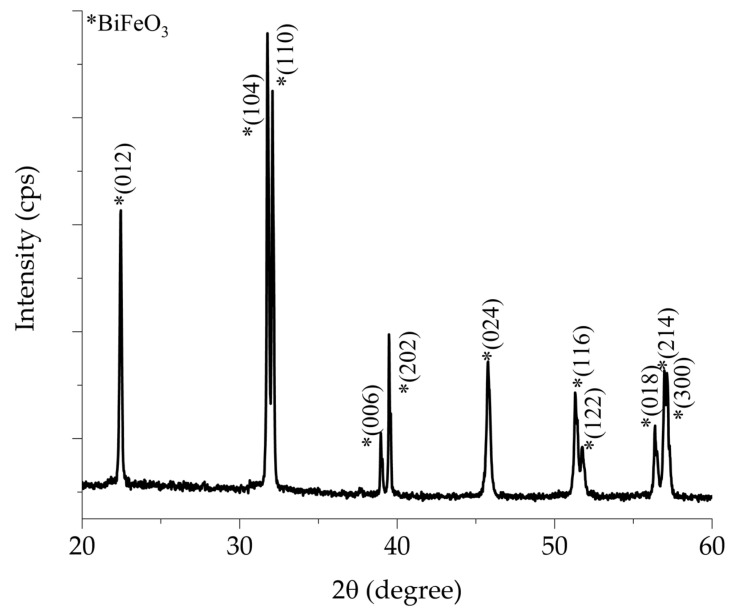
X-ray diffraction pattern of BiFeO_3_ ceramic target sintered at 870 °C for 90 min.

Figure 1 shows the X-ray diffraction pattern of the BiFeO_3_ ceramic target doped with 10% mol of Titanium. The characteristic splitting associated with the rhombohedral phase can be observed within the 2θ angles of 31.77° to 32.08° and 38.98° to 39.53°, and they are identified with an asterisk (*). Additionally, there is a reflection at 37.66°, which confirms the formation of the rhombohedral BiFeO_3_ phase with the space group *R3c*. These reflections have been indexed according to crystallographic card COD #96-450-1316. Secondary phase reflections are not observed. Bismuth ferrite is known to be highly susceptible to the formation of secondary phases, such as Bi_25_FeO_40_, which can occur due to an excess of bismuth in the raw materials [5,24]. Additionally, significant thermal instability has been reported above 675 °C [6,14] influenced by the kinetics of phase formation and the temperature [24] during target fabrication.

**Figure 2:**
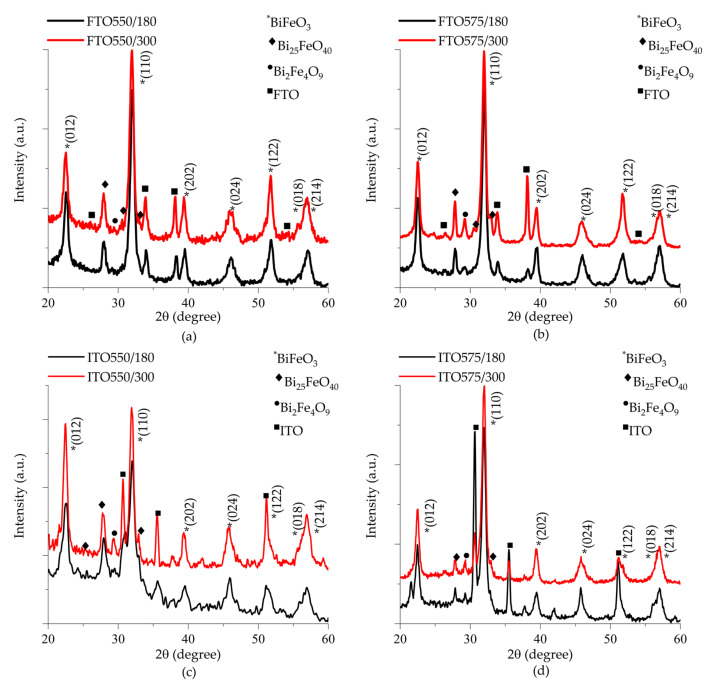
X-ray diffraction patterns of thin films deposited on (**a**,**b**) FTO, and (**c**,**d**) ITO substrate as a function of deposition time and heat treatment temperature.

Figure 2 shows the X-ray diffraction patterns of thin films deposited on (a) FTO and (c) ITO substrates at 550 °C, as well as (b) FTO and (d) ITO substrates at 575 °C. Reflections observed at 22.40°, 32.02°, 39.45°, 45.73°, 51.29°, 57.10°, and 57.31° in 2θ angles correspond to the BiFeO_3_ phase, identified with an asterisk (*). The well-defined peaks indicate high crystallinity, with additional reflections attributed to the FTO substrate in Figure 2a,b and the ITO substrate in Figure 2c,d. Secondary phases are also presented in all samples, including an iron-rich Bi_2_Fe_4_O_9_ phase (mullite structure, Pbam space group, COD #96-900-8149) and a bismuth-rich Bi_25_FeO_40_ phase (selenite structure, I23 space group, COD #96-901-1269). In all the diffraction patterns, the mullite structure is identified with a circle (^•^), and the selenite structure with a diamond (^♦^). The intensity of these secondary phase reflections increases with deposition time for samples deposited on FTO substrates. It is attributed specifically to a destabilization of the secondary phases with the interaction of argon ions during the cathodic erosion process. Meanwhile, the secondary phase reflection intensity remains unchanged for the samples deposited on ITO substrates.

The mechanism of crystallization secondary phases in bismuth ferrite is linked to stoichiometric imbalance, which causes uneven growth of FeO monomers. This imbalance can result in the evaporation of some particles during crystallization, leading to side reactions and the formation of impurity phases [25]. According to Bea et al. iron-rich phases form under low-pressure conditions and temperatures close to 560 °C. Bismuth-rich phases are generated at temperatures ranging from 520 to 560 °C and high-pressure conditions [26]. Both phases are slightly more thermodynamically stable than BiFeO_3_ in the sintered temperatures range between 447 to 767 °C [27].

### 3.2. SEM

#### 3.2.1. Morphology

**Figure 3:**
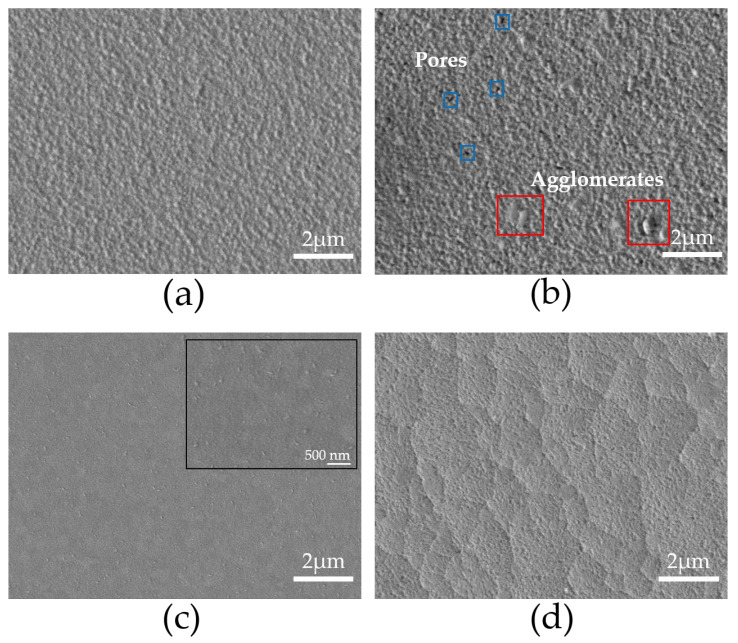
SEM images of the surface morphology of the samples (**a**) FTO550/180, (**b**) FTO575/180, (**c**) ITO550/300, and (**d**) ITO575/300.

Figure 3 illustrates the surface morphology of thin films deposited on FTO and ITO substrates at temperatures 550 °C and 575 °C, using deposition times of 180 and 300 min. The FTO550/180 sample is shown in Figure 3a. The surface is homogeneous, featuring grains that range in size from 114 nm to 176 nm, and is free from pores or cracks. Upon increasing the heat treatment temperature to 575 °C (sample FTO575/180), the grain size remains similar, ranging from 114 to 197 nm. However, at this temperature, pores of approximately 66 nm and agglomerates measuring between 363 to 387 nm begin to appear, as shown in Figure 3b. Additionally, the morphology becomes more irregular, resulting in visible surface defects. At 550 °C, the ITO550/300 sample displays a smooth and compact surface, featuring bright agglomerates that are about 99 nm in size, as illustrated in Figure 3c. This phenomenon is attributed to the precipitation of bismuth on the surface of the thin film [26]. No pores or cracks are present in this sample. At a heat treatment temperature of 575 °C, the ITO575/300 sample shows the formation of pores with an average size of 19 nm, while no precipitates are detected, as shown in Figure 3d. The increase in temperature during the treatment has a significant impact on the morphology of thin films on ITO, causing a change from a smooth, compact surface to one characterized by pores. In contrast, both pores and agglomerates are observed on FTO surfaces.

#### 3.2.2. Thickness

**Figure 4:**
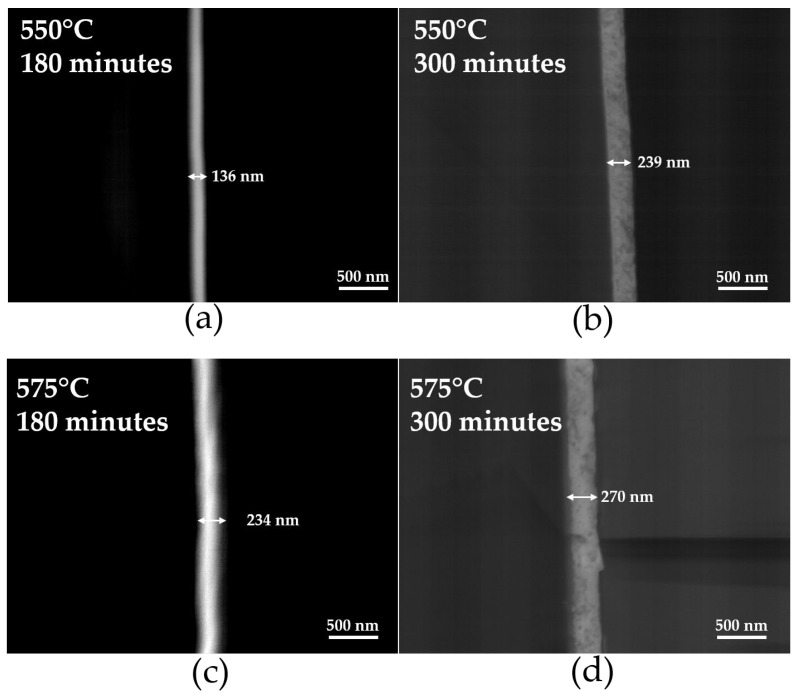
SEM images of the cross-section of Ti-doped BiFeO_3_ thin films deposited on a glass substrate after heat treatment 550 °C: (**a**) 180 min, (**b**) 300 min, at 575 °C (**c**) 180 min, and (**d**) 300 min.

Table 2 shows the thickness of thin films deposited on a glass substrate as a function of deposition time (180 and 300 min) at 550 and 575 °C. Figure 4 presents a cross-sectional image of thin films deposited on glass (a) 180, and (b) 300 min at 550 °C, and (c) 180, and (d) 300 min at 575 °C. As the deposition time increases, the thickness gradually grows. At 575 °C, the thickness increases from 234 nm to 270 nm between 180 and 300 min. The thickness of the thin films increases with both the rise in the heat treatment temperature and the increase in deposition time.

### 3.3. AFM

**Figure 5:**
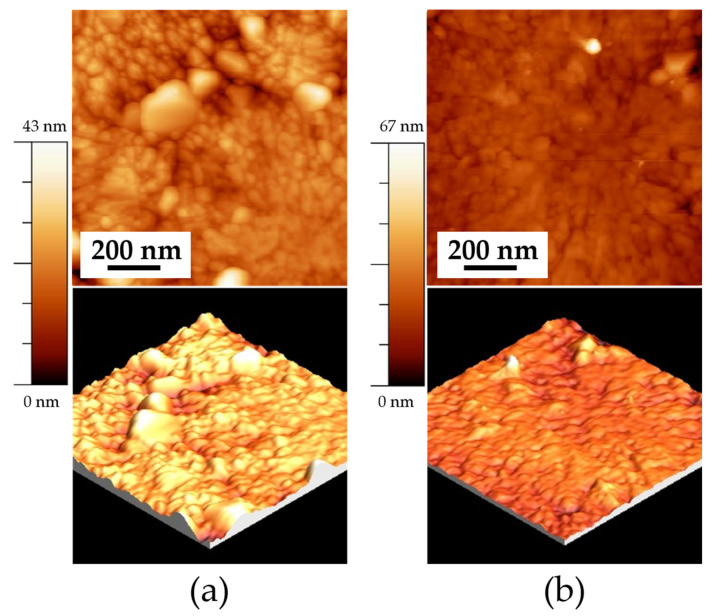
AFM images of the samples deposited on ITO at a heat treatment temperature of 550 °C: (**a**) ITO550/180 (1 × 1 μm) 2D, and 3D, and (**b**) ITO550/300 (1 × 1 μm) 2D and 3D.

Figure 5 shows the AFM images revealing the surface microstructure of thin films heat treated at 550 °C. Figure 5a shows the topography of the ITO550/180 sample, with an RMS roughness of 4 ± 1 nm and an average grain size of 79 ± 0.5 nm. Figure 5b exhibits an RMS roughness of 4 ± 0.5 nm and a 79 ± 1 nm grain size for the ITO550/300 sample. No significant differences in grain size are observed between 180 and 300 min of deposition time. The three-dimensional image (bottom) reveals an irregular growth pattern with the formation of three-dimensional islands, which suggests a Volmer–Weber growth mechanism. This growth mechanism occurs when the interaction between the deposited atoms is stronger than their adhesion to the substrate, which promotes nucleation and the growth of separate islands rather than a uniform layer. As a result, the film exhibits a rough and discontinuous morphology. This type of growth is typical in systems where there is poor wetting of the substrate, such as metals deposited on oxides or semiconductors [11,28,29].

**Figure 6:**
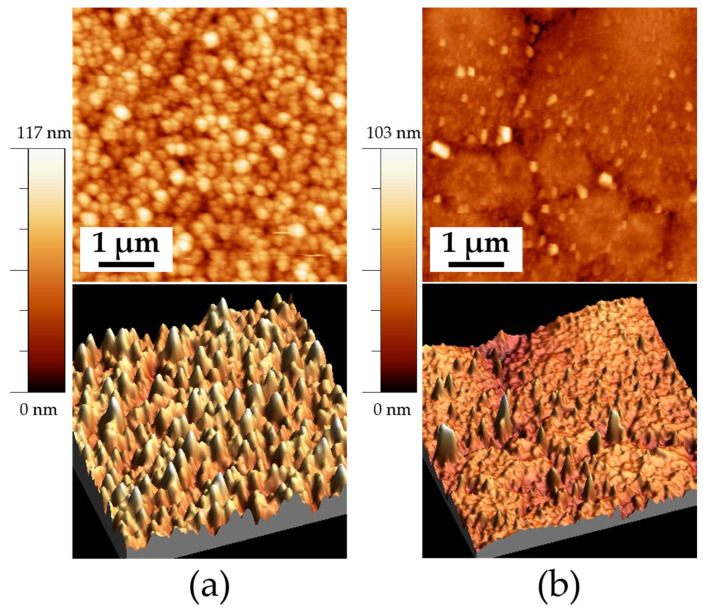
AFM images of the samples deposited on ITO and FTO at a heat temperature of 575 °C: (**a**) FTO575/300 (5 × 5 μm) 2D, and 3D and (**b**) ITO575/300 (5 × 5 μm) 2D and 3D.

Figure 6 shows the morphology of the samples with 300 min of deposition and a heat treatment temperature of 575 °C. A significant difference in grain formation and growth is observed when comparing both samples. The sample deposited on FTO, shown in Figure 6a, exhibits a more homogeneous morphology, characterized by spherical grains with an average size of 185 ± 1 nm. Additionally, this sample presents an RMS roughness of 13 ± 1 nm. In contrast, the sample deposited on ITO (ITO575/300) shows brighter agglomerates at the grain boundaries, consistent with the SEM images. This sample exhibits an RMS roughness of 6 ± 0.5 nm.

The growth of thin films is consistent with the observations reported by Zhu et al., who described grain growth as islands with fine and distinct vertical grains, along with surface roughness and voids between grains when thin films are deposited on FTO. However, the voids created by this island growth tend to form conductive channels when an electric field is applied, increasing leakage current and dielectric loss [30].

### 3.4. PFM

**Figure 7:**
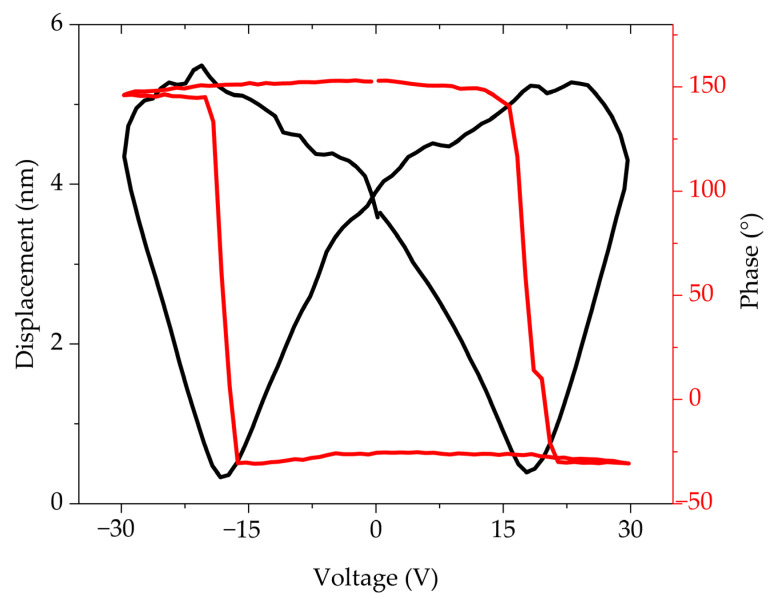
PFM amplitude–voltage butterfly loop (black) and phase–voltage hysteresis loop (red) of FTO575/300 thin film.

The local piezo-ferroelectric response of the samples was studied by SSPFM-DART mode to obtain the amplitude (displacement) and phase plots in the “Off” state, These measurements are normally presented in this state since this eliminates the long-range electrostatic force influencing the electromechanical response on the samples. Figure 7 shows the plot for the FTO575/300 sample of the amplitude response showing the typical “butterfly” behavior of ferroelectric materials. A d_33_ value of 2.71 nm/V was obtained from the displacement versus voltage plot, meanwhile, the switching of the ferroelectric domains is observed in the phase plot, confirming the local switching of the domains and corroborating the local piezoresponse of the sample. To compare the effect of the substrate, sample ITO575/300 was studied by the SSPFM-DART mode. The obtained results showed poor piezo-ferroelectric response from the sample, leading to anomalous results in both the displacement and phase plots. This could be attributed to a poor-quality sample or the diffusion of ITO into the thin film as can be observed in Figure 6a.

### 3.5. Optical Properties

#### 3.5.1. Absorption Spectra

**Figure 8:**
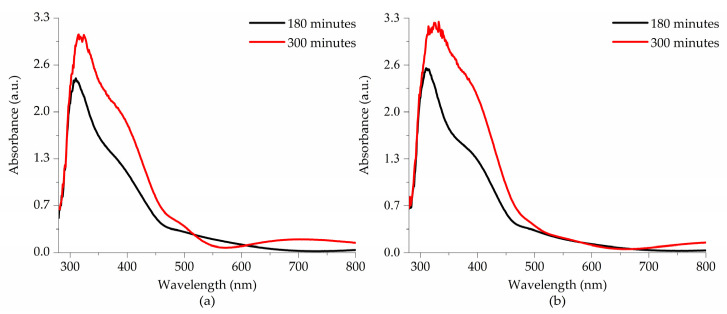
Absorption spectra of the thin films subjected to thermal treatment at (**a**) 550 °C and (**b**) 575 °C.

Figure 8 presents the absorbance spectra of thin films deposited on glass substrates and subjected to thermal treatment at (a) 550 °C and (b) 575 °C as a function of deposition time. A clear trend of increasing absorbance values is observed with longer deposition times, which is attributed to the increased thickness of the thin films. Additionally, a slight enhancement in absorbance intensity is observed when the thermal treatment temperature is raised from 550 °C to 575 °C. These absorption values obtained in the thin films correspond to an average absorption coefficient of 1.88 × 10^5^ cm^−1^, ranging from 280 to 480 nm.

All spectra exhibit three absorption edges. The first and most intense is around 320 nm, corresponding to interatomic transitions involving charge transfer from the O 2p states in the valence band to the Fe 3d states in the conduction band. The second absorption edge, around 410 nm, is attributed to charge transfer transitions between Fe1 3d and Fe2 3d sites. Finally, a third absorption edge, with much lower intensity, appears at approximately 688 nm. This band is associated with 6A1g → 4T2g transitions, corresponding to d-d transitions in Fe^3+^ ions. Although these excitations are formally forbidden, they exhibit low intensity due to the relaxation of the selection rule caused by spin–orbit coupling [7,31].

#### 3.5.2. Bandgap

The band gap was estimated using the Tauc method for directly allowed transitions. It was determined by plotting (αhν)^2^ versus the energy hν. By extrapolating the linear portion of the graph to the point where α = 0, we can estimate the value of the band gap.

**Figure 9:**
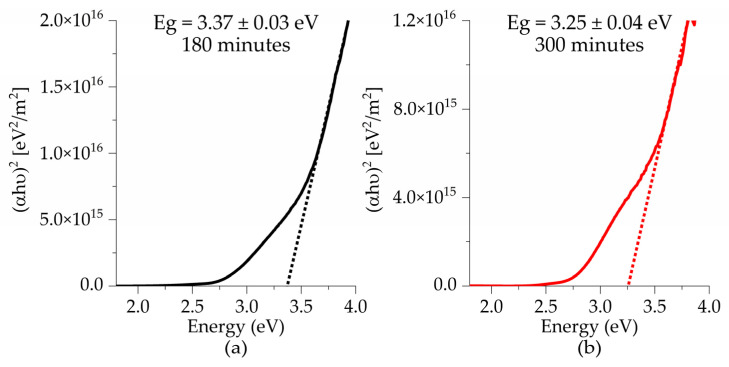

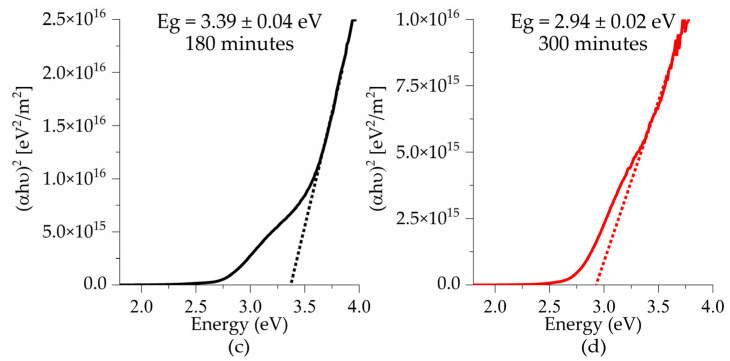
Tauc plots of BFTO thin films as a function of deposition times (**a**,**b**) 550 °C and (**c**,**d**) 575 °C.

Figure 9 presents the band gap values for all samples deposited on glass substrates as a function of deposition time. A decrease in the band gap is observed as the deposition time increases. For films treated at 575 °C, the obtained values were 3.39 eV at 180 min, and 2.94 eV at 300 min, which is consistent with the findings reported by Sando et al., who attributed this trend to the increase in film thickness due to changes in the microstructure and a reduction in surface defects, both of which contribute to a decrease in the values of the band gap [29]. As the thermal treatment temperature increases from 550 to 575 °C, a decrease in the band gap from 3.25 eV to 2.94 eV is observed for samples with a deposition time of 300 min. This can be attributed to an improvement in the crystallization process, which results in a more ordered atomic arrangement, thereby narrowing the band gap [32].

Also, the decrease in the band gap can be attributed to defects in the thin films. Defects such as oxygen vacancies induced by the addition of titanium lead to the formation of Fe^+3^ and Fe^+4^ valences [4,12,16]. These defects generate donor levels below the conduction band, which contribute to the reduction of the band gap. Furthermore, bismuth vacancies formed during sintering or thermal treatment processes create acceptor levels above the valence band, causing a slight further decrease in the band gap [15,33].

### 3.6. Ferroelectric Properties

**Figure 10:**
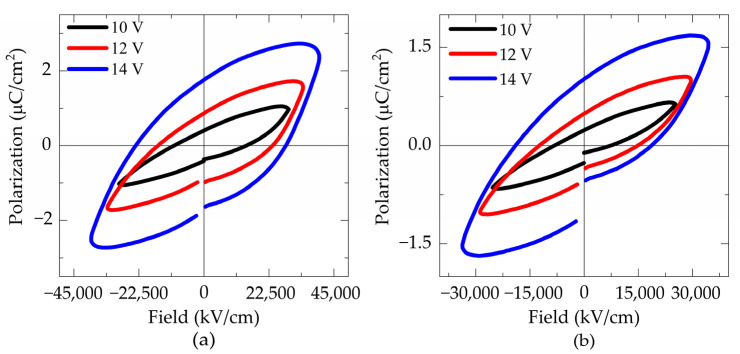

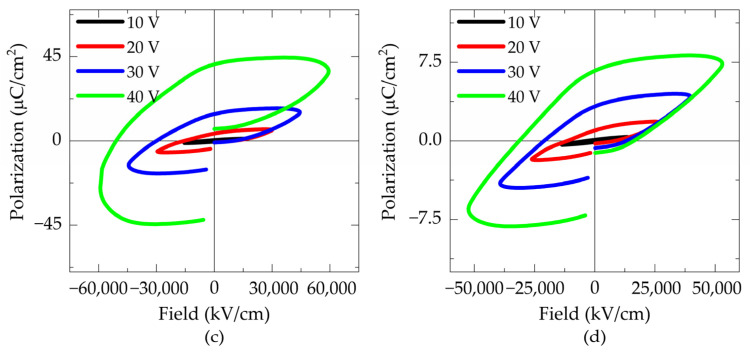
P-E hysteresis loops measured at 10 kHz of the samples: (**a**) FTO550/180, (**b**) FTO575/180, (**c**) ITO550/300, and (**d**) ITO575/300.

Figure 10 shows the hysteresis loop of (a) FTO550/180 and (b) FTO575/180 samples. As presented in Table 3 increasing the heat treatment temperature from 550 °C to 575 °C results in a decrease in the maximum saturation polarization from 2.708 to 1.692 μC/cm^2^, while the remanent polarization remains similar about 1.746 μC/cm^2^ and 1.045 μC/cm^2^ for FTO550/180 and FTO575/180 samples, respectively. Remanent polarization is strongly dependent on the pure phase [13] and can be correlated with the X-ray diffraction patterns. The intensity of the Bi_2_Fe_4_O_9_ peaks increases at higher temperatures for thin films deposited on FTO.

Figure 10c,d present the hysteresis loops of thin films deposited for 300 min on ITO. Both ITO550/300 and ITO575/300 films exhibit similar loop shapes. The film treated at 550 °C achieved a maximum saturation polarization of 40.89 μC/cm^2^ and a remanent polarization of 44.87 μC/cm^2^, representing the highest values obtained. In contrast, the film treated at 575 °C exhibited a maximum polarization of 8.45 μC/cm^2^ and a remanent polarization of 6.68 μC/cm^2^.

None of the hysteresis loops obtained shows clear saturation. Instead, rounded loops with noticeable leakage currents are observed. This leakage can be attributed to several factors, including the addition of titanium to the starting stoichiometry, which induces valence changes in Fe^2+^ and Fe^3+^, leading to the formation of oxygen vacancies. These vacancies create shallow energy centers, a common issue in bismuth ferrite, which could limit its potential for photovoltaic applications [16]. The poor ferroelectric properties can also be attributed to the presence of the Bi_2_Fe_4_O_9_ phase, which contributes to charge defects that increase leakage current [34]. A third cause may be the formation of pores and cracks, resulting in high leakage due to the lower dielectric constant of the air within them [35]. Additionally, Volmer–Weber (VW) type grain growth produces voids between grains (pores and cracks) as a result of three-dimensional island growth, which tends to open conductive channels under an applied electric field, leading to increased leakage current and dielectric loss [30].

## 4. Conclusions

In this work, Ti-doped BiFeO_3_ thin films were obtained from a homemade target. The majority phase in both the target and the thin films is rhombohedral BiFeO_3_. However, after sputtering the target, secondary phases (Bi_25_FeO_40_ and Bi_2_Fe_4_O_9_) were detected in all the thin films. This is attributed to the impact of argon and oxygen ions, which lead to the formation of these secondary phases. The local piezo-ferroelectric response of the thin films deposited on FTO substrates shows the amplitude response obtained from the typical “butterfly” behavior of ferroelectric materials and a d_33_ value of 2.71 nm/V. While thin film was deposited on ITO substrates, it was not possible to obtain this behavior due to several defects. We attribute this to the formation of islands via the Volmer–Weber growth mechanism. This does not allow adequate contact between the tip and the film surface. The films deposited on ITO achieved a maximum saturation polarization of 40.89 μC/cm^2^ and a remanent polarization of 44.87 μC/cm^2^, representing the highest values obtained. However, as the grain size increased, charge defects led to a higher leakage current. This is attributed to the formation of three-dimensional island grains, which create voids that, when an electric field is applied, lead to the formation of conductive channels, thereby increasing the leakage current. The films exhibit an absorption coefficient of 1.88 × 10^5^ cm^−1^ ranging from 280 to 480 nm. Additionally, a reduction of the bandgap to less than 3 eV was achieved. Despite the presence of secondary phases in the thin films, they exhibit good optical properties and a high remanent polarization, features that are favorable for potential application in solar cells as an absorbing material, leveraging the ferroelectric photovoltaic effect.

## Figures and Tables

**Table 1:** Experimental conditions of thin film samples deposited on ITO and FTO.

Sample	Substrate	Heat Treatment Temperature [°C]	Deposition Time [min]
ITO550/180	ITO	550	180
ITO575/180	ITO	575	180
ITO550/300	ITO	550	300
ITO575/300	ITO	575	300
FTO550/180	FTO	550	180
FTO575/180	FTO	575	180
FTO550/300	FTO	550	300
FTO575/300	FTO	575	300

**Table 2:** Comparison of the thickness of thin films deposited on a glass substrate at heat treatment temperatures of 550 °C and 575 °C as a function of deposition time.

550 °C		575 °C	
Deposition Time [min]	Thickness [nm]	Deposition Time [min]	Thickness [nm]
180	136 ± 14	180	234 ± 16
300	239 ± 11	300	270 ± 17

**Table 3:** Ferroelectric data of thin films deposited on ITO and FTO substrates.

Sample	Maximum Polarization	Remanent Polarization
	[µC/cm^2^]	[µC/cm^2^]
ITO550/300	40.89	44.87
ITO575/300	8.45	6.68
FTO550/180	2.708	1.746
FTO550/300	0.234	0.0049
FTO575/180	1.692	1.045
FTO575/300	0.241	0.0011

## Data Availability

The original contributions presented in this study are included in the article. Further inquiries can be directed to the corresponding authors.
